# Distinct effects of mucin on phage-host interactions in model systems of beneficial and pathogenic bacteria

**DOI:** 10.1007/s00705-025-06322-5

**Published:** 2025-05-20

**Authors:** Jaka Jakin Lazar, Katarina Šimunović, Iztok Dogša, Ines Mandić Mulec, Mathias Middelboe, Anna Dragoš

**Affiliations:** 1 Department of Microbiology, Biotechnical Faculty, University of Ljubljana, Ljubljana, Slovenia; 2 Department of Biology, University of Copenhagen, Copenhagen, Denmark; 3 Department of Biology, University of Southern Denmark, Odense, Denmark

## Abstract

Phage-host interactions that occur in host-associated microbiomes are influenced by a plethora of environmental factors. Mucins are glycoproteins that represent the main component of mucus, which is found in the animal digestive tract and on the surface of certain organs, serving as the first line of defense against toxins and pathogens. Previous studies have shown that lytic phages have an important influence on the microbial composition in mucosal areas. Our study expands this knowledge to interactions between previously untested lytic phages targeting probiotic and pathogenic bacteria, as well as temperate phages targeting probiotic bacteria. These interactions could be important in shaping microbial communities and affecting the well-being of their host. This study demonstrates that mucins enhance the adherence of *Vibrio anguillarum* lytic phages and *Bacillus subtilis* lytic and temperate phages, as well as *B. subtilis* and *V. anguillarum* cells, to solid surfaces. Our results also show that mucins positively affect the attachment of *B. subtilis* cells even in the presence of phages. This positive effect was not observed in the case of *V. anguillarum*. This suggests that mucin may shield certain bacteria from phage infections. We also found that mucin influenced the metabolic activity of the two tested bacterial species differently, with strong positive effects on *V. anguillarum* but not on *B. subtilis*. This work supports previous findings that phages adhere efficiently to mucus and extends these studies to include other beneficial and pathogenic bacterial species. It also reveals that mucins have different effects on phage-host interactions in different phage-host systems, which may have implications for phage therapies or probiotic treatment strategies.

**Supplementary Information:**

The online version contains supplementary material available at 10.1007/s00705-025-06322-5.

## Introduction

Interactions between phages and their bacterial hosts are significantly influenced by a range of environmental factors, including physical and chemical conditions [1, 2], nutrient availability [3, 4], the presence of other organisms, and various polymers produced by these organisms [5]. Recent studies have shown that phages can become trapped within the extracellular matrix of bacterial biofilms, which can act as a barrier to phage infection [5–7]. Earlier research has suggested that the mucus layer – a dynamic and complex environment composed of glycoproteins, immune factors, and microbial communities – plays a substantial role in host-phage interactions [8].

Mucus lines the surfaces of many organs and tissues in the body, and it is predominantly composed of mucins, which are high-molecular-weight glycoproteins that have diverse functions, including protection against pathogens and toxins and modulation of cell signaling [9]. Mucins also interact with microorganisms by providing attachment sites or inhibiting their growth or virulence [10].

In 2013, Barr et al. introduced the Bacteriophage Adherence to Mucus (BAM) model [8]. This model examines how phages interact with mucin, showing an increased phage-to-bacteria ratio on various mucosal surfaces from cnidarians to humans. The increased adherence of phages to mucus protects underlying tissue from bacterial infection by facilitating phage infection of mucosal bacteria, thus providing a form of non-host-derived immunity. Research has focused on the binding of mucin glycoproteins and Ig-like protein domains on phage capsids [8]. Initially focused on *E. coli* phage T4, subsequent research studies have extended the BAM model’s applicability to diverse phages such as *Flavobacterium columnare* phage FCL-2, *Aeromonas* sp. phage V46, *Flavobacterium* sp. phage FL-1, and *Pseudomonas aeruginosa* phages GEC_MRC, GEC_K2, and GEC_PNG3. These phages also contain Ig-like domains on their capsids [11, 12]. On the other hand, it has been shown that *E. coli* phage ES17 can adhere to mucin through its tail fiber protein [13]. While most studies have focused on phages targeting Gram-negative pathogens, evidence suggests that mucin promotes phage adherence across a broad range of phages with diverse structural components [11–14].

Mucin has also been shown to enhance the adherence of certain bacteria [15]. However, its effects on phage-host interaction of different species, such as probiotics and pathogens, which could potentially coexist within a eukaryotic host, have not been compared directly. As a first step toward a better understanding of the ability of the mucin environment to modulate bacterial multispecies communities, we focused on two unrelated species with contrasting biomedical relevance: the probiotic *Bacillus subtilis* and the pathogenic *Vibrio anguillarum*.

*B. subtilis* is an example of a beneficial Gram-positive bacterium that can be found in various environments, such as food, soil, and animal gut, where it is also used as a probiotic [16]. *V. anguillarum* is an important fish-pathogenic, Gram-negative bacterium that is found in marine environments and causes vibriosis [17], a major disease that causes problems in aquaculture facilities [18].

Prior studies have demonstrated that certain strains of *B. subtilis* can adhere to epithelial colon tissue Caco-2 cells, while none of the tested strains could attach to the cell line HT29-16E, which displays differentiation features that are characteristic of mature intestinal cells. Notably, HT29-16E cells are mucin-producing cells, while Caco-2 cells are not significant producers of mucin [19–21]. Zhang et al. [22] conducted a study that demonstrated the ability of *B. subtilis* to adhere to isolated tilapia intestinal mucus in an *in vitro* setting. Another research investigation has demonstrated strain-specific differences in the ability of *B. subtilis* to adhere to mucin [23]. Other studies have shown that *V. anguillarum* has ability to adhere to mucus isolated from fish [24, 25]. However, to our knowledge, direct adherence of *V. anguillarum* to mucins *in vitro* has not been studied so far.

In the current study, we found that phages specific for both species exhibited increased adherence to mucin when compared to control surfaces. Furthermore, their bacterial hosts, *B. subtilis* and *V. anguillarum*, also showed increased adherence to mucin when compared to control surfaces. Our findings further showed that mucin exerts distinct effects on phage-host interactions and metabolic activity during surface attachment of these two bacterial species. While mucin generally influences phage and bacterial adherence to surfaces, our results underscore the importance of examining its effects on each specific phage-host interaction individually.

## Materials and methods

### Bacterial strains, phage stocks, and growth conditions

*B. subtilis* strain Δ6 (accession number NZ_CP015975.1) [26] was used as an indicator strain in all phage adherence experiments. *B. subtilis* strain P9_B1 (accession number CP045811.1) [27] or its fluorescently labeled derivative *B. subtilis* P9_B1 *amyE*::*gfp* were used in bacterial attachment assays [28]. *V. anguillarum* strain PF430-3 (accession number NZ_CP011467) [6, 29] was used in all *V. anguillarum* experiments. The following phages were used where indicated: *B. subtilis* lytic phage Nf (accession number NC_049976) [Shimizu et al., 1970], *B. subtilis* lytic phage SP-10 (accession number NC_019487) [30], *B. subtilis* temperate phage SPβ (accession number NC_000964) [31], and *V. anguillarum* lytic phage KVP40 (accession number AY283928) [32].

*B. subtilis* strains were maintained in lysogeny broth (LB) (LB (Lennox), Laboratorios Conda; 5 g/L yeast extract, 10 g/L tryptone, 5 g/L NaCl, dH_2_O) at 37°C with shaking at 220 rpm. *V. anguillarum* was maintained in marine broth (MB) (5 g/L peptone [Sigma-Aldrich], 1 g/L yeast extract [Sigma-Aldrich], 20 g/L NaCl [Sigma-Aldrich], dH_2_O) for 24 hours at 30 °C with shaking at 220 rpm.

Phages were propagated using a mid-log-phase culture of either *B. subtilis* Δ6 in LB broth or *V. anguillarum* in MB medium. A 50-mL aliquot of host culture was inoculated with phage stock at a multiplicity of infection (MOI) of 10. The mixture was incubated at 37 °C for *B. subtilis* or 30 °C for *V. anguillarum* with shaking at 220 rpm for 16 hours. Lysates were centrifuged at 8000 x *g* for 15 minutes to remove cellular debris and then passed through a 0.22-μm sterile filter to obtain a cell-free phage lysate, which was then stored at 4°C.

### Phage adherence to mucin assay

The phage adherence to mucin assay was performed as described previously [14]. Briefly, LB agar (LB agar (Lennox), Laboratorios Conda; 5 g/L yeast extract, 10 g/L tryptone, 5 g/L NaCl, 15 g/L bacteriological agar, dH_2_O) or marine broth (MB) agar plates (5 g/L peptone [Sigma-Aldrich], 1g/L yeast extract [Sigma-Aldrich], 20 g/L NaCl [Sigma-Aldrich], and 15 g/L agar [Sigma-Aldrich]) were coated with either 1 mL of 1% (w/v) type III porcine stomach mucin (Sigma-Aldrich) in 1X phosphate-buffered saline (PBS) or 1 mL of 1% (w/v) bovine serum albumin (BSA) (Sigma-Aldrich) in 1X PBS and then left at room temperature until the liquid was no longer visible. Some plates were left uncoated. Stocks of phages were serially diluted in LB to a concentration of 10^2^ PFU/mL. Next, 5-mL aliquots of diluted phage stocks were transferred to the coated and uncoated plates, which were then incubated for 1 hour at 37 °C on an orbital shaker at 25 rpm. Afterwards, the phage suspensions were decanted from the plates, and the plates were covered with 100 µL of exponentially growing *B. subtilis* Δ6 or *V. anguillarum* PF430-3 resuspended in soft LB agar (5 g/L yeast extract, 10 g/L tryptone, 5 g/L NaCl, 3 g/L agar, dH_2_O) or soft MB agar (5 g/L peptone [Sigma-Aldrich], 1 g/L yeast extract [Sigma-Aldrich], 20 g/L NaCl [Sigma-Aldrich], and 3 g/L agar [Sigma-Aldrich]). The plates were then incubated overnight at 28 °C, and the plaques were counted the next day. The number of phages that adhered to mucin was calculated using the dilution factor.

We used type III porcine stomach mucin (Sigma-Aldrich) due to its standardized production process, ensuring consistency across experiments. This mucin type is also widely available and cost-effective, making it a commonly used choice for *in vitro* studies. It has been used previously in research examining phage and bacterial interactions with mucins [14].

### Bacterial adherence to mucin assay

The effect of mucin on *B. subtilis* cell attachment was quantified by both confocal laser scanning microscopy and flow cytometry, whereas counting of attached *V. anguillarum* cells was performed by flow cytometry only. The effect of phages on bacterial attachment was also tested using flow cytometry.

### Confocal laser scanning microscopy

Prior to imaging, 1.5% agar plates (Agar, Laboratorios Conda; 15 g/L agar, dH_2_O) were coated with either 1 mL of 1% w/v porcine stomach mucin type III in 1x PBS or 1 mL of 1% w/v BSA in 1x PBS and then allowed to dry. Some of the plates were left uncoated. *B. subtilis* P9_B1 *amyE*::*gfp* cultures were incubated in LBGM (5 g/L yeast extract, 10 g/L tryptone, 5 g/L NaCl, 10 mL/L 100% glycerol [Sigma-Aldrich], 1 mL/L MnSO_4_ [stock concentration 100 mM, Sigma-Aldrich], dH_2_O) for 24 hours at 37 °C with shaking at 220 rpm and then diluted by a factor of 10. Next, 5-mL aliquots of this diluted culture were transferred to mucin-coated and uncoated plates, which were then incubated for 1 hour at 37 °C on an orbital shaker at 25 rpm. Afterwards, the bacterial suspensions were decanted from the plates, followed by two washing steps with 5 mL of 1x PBS. The plates were dried, and a piece of agar from the center of each plate was cut out and placed upside down on a glass microscope slide. Finally, the samples were visualized using an inverted confocal laser scanning microscope (AxioObserver Z1, LSM800) (Zeiss) using a 20×/0.4 EC Plan-Neofluar objective. Transmitted light was acquired simultaneously with the fluorescent confocal channel using T-PMT and GaAsP PMT, respectively. The settings used for imaging were as follows: green fluorescence laser wavelength, 488 nm; power, 1%; pinhole size, 1.76 AU/84 μm; detector gain, 750 V; image size (pixels), 1073 px × 1073 px; pixel time, 1.97 μs. The images were analyzed using Fiji (ImageJ) software [33] (https://imagej.net/software/fiji/). Attached cells visible in recorded images were counted using a recorded macro (Supplemental file 1) with the “Adjust Threshold” and “Analyze Particles” functions. The experiment was performed in triplicate, with five technical replicates for each surface (five captured images per agar surface). The surface area visualized in each image was 0.1 mm^2^. The number of cells was multiplied by 418.5 to obtain the number of cells per 41.85 mm^2^, which was the surface area used for cell counting using the flow cytometer in the protocol below.

### Flow cytometry

Prior to flow cytometry experiments, 1.5% agar plates were coated with 1 mL of 1% w/v porcine stomach mucin type III in 1x PBS, 1 mL of 10^7^ PFU/mL phage suspension (Nf or KVP40), or 1 mL of 1x PBS and then allowed to dry. Next, 5 mL of phage suspension (Nf for *B. subtilis* or KVP40 for *V. anguillarum*) with a titer of ~10^7^ was transferred to the mucin-coated plates, which were then incubated for 1 hour at 37 °C on an orbital shaker at 25 rpm, and the phage suspensions were decanted from these plates.

*B. subtilis* P9_B1 cultures were incubated in LBGM for 24 hours at 37 °C with shaking at 220 rpm, and *V. anguillarum* PF430-3 cultures were incubated in marine broth (MB) (5 g/L peptone [Sigma-Aldrich], 1 g/L yeast extract [Sigma-Aldrich], 20 g/L NaCl [Sigma-Aldrich]) for 24 hours at 30 °C with shaking at 220 rpm. Both cultures were then diluted by a factor of 10. Next, 5-mL aliquots of the diluted *B. subtilis* and *V. anguillarum* cultures were transferred to the coated and uncoated plates, which were then incubated for 6 hours at 37 °C (*B. subtilis*) or 30 °C (*V. anguillarum*) on an orbital shaker at 25 rpm. Afterwards, the bacterial suspensions were decanted from the plates, followed by two washing steps with 1x PBS. The plates were dried, and a piece of agar was cut out of each plate using the top end of a pipette tip.

The diameter of the top end of the pipette tip is 7.3 mm, so the cut surface area was calculated to be 41.85 mm^2^. The piece of agar was then transferred to a microcentrifuge tube containing 1 mL of saline solution, and after mixing on a vortex stirrer for 15 seconds, and the bacterial cells were detached from the agar pieces by sonication three times for 15 seconds each in an ultrasonic bath with intermittent mixing on a vortex stirrer. The tubes were then centrifuged for 15 minutes at 800 × *g*. As a result, the agar, which had been broken down during ultrasonication, formed a pellet at the bottom, while some of the cells remained in the supernatant. The supernatant was carefully transferred to new microcentrifuge tubes, and 10 μL of 25% glutaraldehyde (Sigma-Aldrich) was added to these tubes to fix the cells in the solution.

Fixed cells were counted using a flow cytometer (BD Biosciences) as follows: 50 μL of the fixed cell suspension was diluted in 450 μL of 1X TE buffer (Tris-EDTA; 1 mL 10x Tris-EDTA [Sigma-Aldrich], and 99 mL dH_2_O), after which 5 μL of SYBR Gold stain (Thermo Fisher Scientific) was added. The mixture was then incubated for 10 minutes at room temperature and analyzed using a FACS Melody flow cytometer (Becton Dickinson). Apart from the fixed cells, an overnight bacterial culture was used, which was also diluted in 1X TE buffer and stained as a positive control, and the same culture was left unstained as a negative control. To enable quantitation of the cells, AccuCheck Counting Beads (Thermo Fisher Scientific) were also diluted and analyzed using the same procedure as for the cells. All of the samples were analyzed for 30 seconds at a flow rate of 100, using a blue laser with a wavelength of 488 nm. The bandpass filter used was 527/32, and the long-pass filter used was 507 LP. The data were then processed using FlowJo software (https://www.flowjo.com/), and number of cells per area was calculated using formula “Number of cells/Cut surface area = A/C x B/D”, where A is the concentration of the beads in the counting beads standard, B is the number of events detected for the sample, C is the number of events detected for the counting beads standard, and D is the final dilution factor of the cells.

Graphs were created by plotting the logarithm of the forward scatter (FSC) values on the *y*-axis and the logarithm of the green fluorescence intensity on the *x*-axis. Positive and negative controls (stained and unstained bacterial cultures, respectively) were used to select the portion of the plot for counting. Each of the *B. subtilis* samples was analyzed in three replicates, while *V. anguillarum* samples were analyzed in six replicates.

### Oxygen consumption measurements

To measure the oxygen consumption rate of bacteria attached to different surfaces, the samples were prepared, and a piece of agar containing attached biofilm was cut out as described above. The samples were placed in a gas-tight vial with an optode patch mounted inside (Presens, Germany), and the vials were filled with either LB for *B. subtilis* or MB for *V. anguillarum* samples, leaving no air space. The vials and the media were pre-heated to 37 °C for *B. subtilis* and 30 °C for *V. anguillarum*. Then, the tubes were placed on a 24-channel oxygen meter (SensorDish reader SDR2, PreSens) [34], which was mounted on a rotating table in an incubator at 37 °C for *B. subtilis* and 30 °C for *V. anguillarum*. For 20 hours, the oxygen concentration was measured every 15 seconds by excitation of the optodes after calibration with 100% and 0% oxygen. The data were collected by a connected computer using PreSens Measurement Studio 2 (https://www.presens.de/products/detail/presens-measurement-studio-2). The data were then exported and analyzed in Microsoft Excel and OriginPro 2024 (https://www.originlab.com/origin). The bacterial respiration rate was calculated from the slope of the initial linear decrease in oxygen concentration over time.

### Colonization assay

To measure the colonization of fresh growth medium, samples were first prepared as described above, and after incubation in SDR SensorDish vials, the liquid content was diluted tenfold in saline solution (9 g/L NaCl) to a final volume of 1 mL. Subsequently, 10 μL of 25% glutaraldehyde was added for fixation, and 50 μL of the sample was mixed with 450 μL of TE buffer and stained with 5 μL of SYBR Gold stain. The stained samples were incubated at room temperature for 10 minutes, and cells were counted by flow cytometry as described above.

### Bioinformatic search of Ig-like domains

To identify potential Ig-like domains in the phage genome sequences used in this study, we used the PHROGS (Phage Orthologous Groups) database to identify genes encoding head and packaging proteins [35], and the SMART web tool was used to identify Ig-like domains in the encoded proteins [36].

### Analysis of phage adherence to mucin

Nf phage adherence to mucin was assessed in three independent experiments, using six technical replicates per surface. Eighteen surfaces were examined over three days, each time with a fresh indicator strain, and the data from different days were normalized. SP10 phage adherence to mucin was assessed in two independent experiments, using five technical replicates per surface. SPꞵ phage adherence to mucin was assessed in two independent experiments, using 18 replicates in total. KVP40 phage adherence to mucin was assessed in one experiment using three replicates per surface. The number of attached phages was calculated for each experiment and presented using box plots, created in OriginPro 2024. In these plots, the whiskers represent 1.5 times the interquartile range, the midline represents the median, and the mean value is indicated by a triangle. In addition, the values obtained from all independent experiments were normalized to the maximum (within a single experiment), averaged, and combined in a single box plot chart.

### Analysis of metabolic activity of bacterial cells attached to different surfaces

Data on oxygen consumption were collected using PreSens Measurement Studio 2 software and visualized in Microsoft Excel. The amount of oxygen in the suspension (in μmol/L) was plotted on the *y*-axis, and time (minutes) was plotted on the *x*-axis. The area on the chart with the initial linear decline in the slope (oxygen amount), was selected and used for linear regression analysis to determine the oxygen consumption rate.

### Statistical analysis

To compare differences between treatments (types of surfaces), a one-way analysis of variance (ANOVA) test was used, followed by the Tukey test. Box plots were created using OriginPro 2024.

## Results

### Mucin promotes surface adherence of *Bacillus *and *Vibrio* phages

Previous studies have demonstrated that mucin coating can promote adherence of certain virulent phages of Gram-negative bacteria to solid surfaces. Here, we quantified the effects of mucin on the attachment of selected virulent and temperate *B. subtilis* phages as well as a virulent *V. anguillarum* phage to surfaces.

**Fig. 1:**
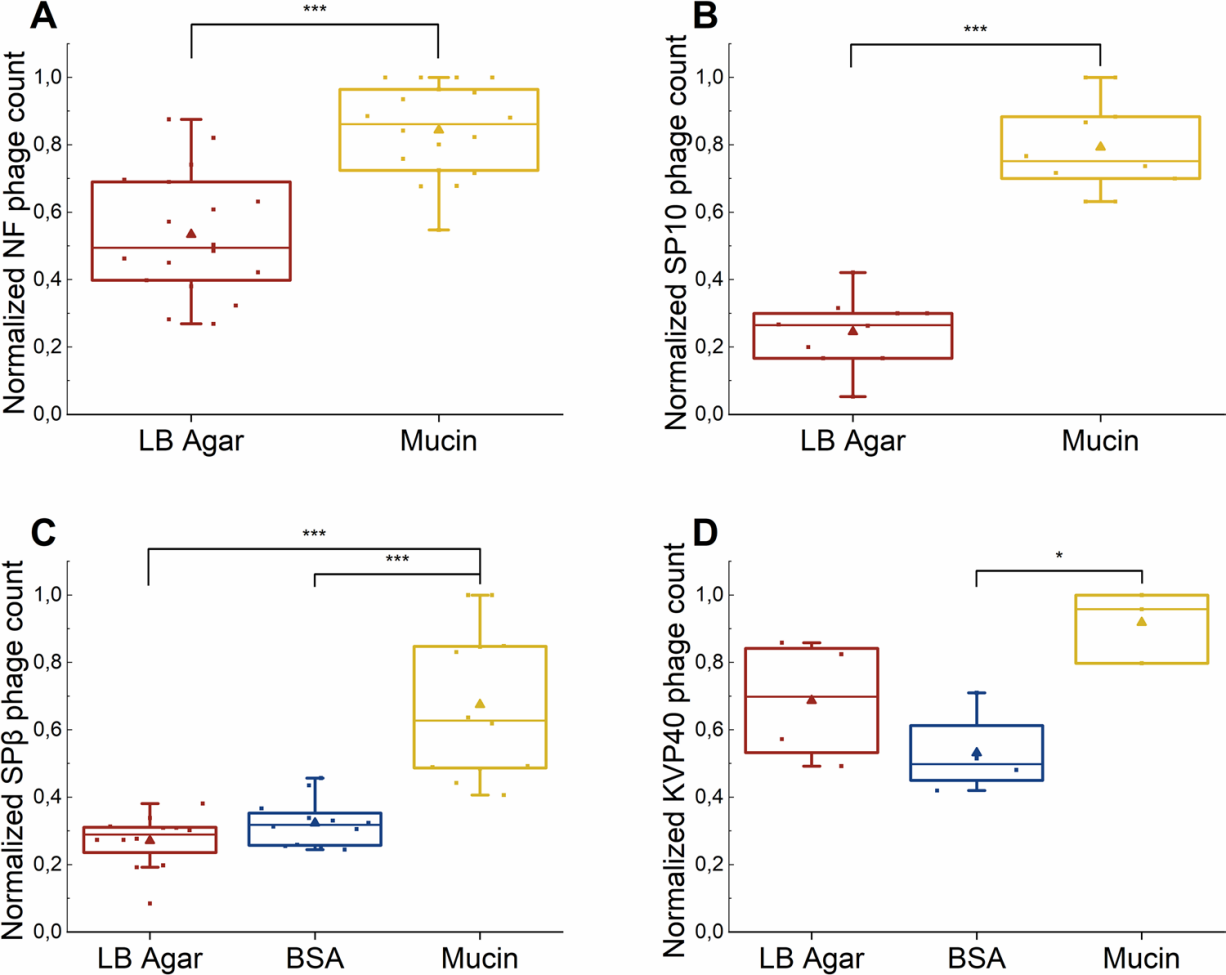
Attachment of phages Nf, SP10, SPꞵ, and KVP40 to mucin. (A) Attachment of phage Nf to mucin compared to control surface (LB agar) (n = 18). (B) Attachment of phage SP10 to mucin compared to control surface (LB agar) (n = 10). (C) Attachment of phage SPꞵ to mucin compared to control surfaces (LB agar and BSA) (n = 12). (D) Attachment of phage KVP40 to mucin compared to control surfaces (LB agar and BSA) (n = 4). Values are normalized to the maximum within a single experiment. Statistical analysis was performed using ANOVA with Tukey’s post hoc test (*, *p* < 0.05; ***, *p* < 0.001).

The data demonstrated that, for all four *Bacillus* phages tested, a significantly larger number of particles bound to mucin-coated surfaces than to LB agar or surfaces coated with BSA (Fig. 1).

Coating with mucin resulted in a 1.3- to 3.2-fold increase in adherence, which is in agreement with previous studies [12, 14]. The data from individual experiments, showing plaque counts prior to normalization, are shown in Supplementary Fig. S1.

### Mucin promotes surface adherence of *B. subtilis* and *V. anguillarum* cells

A previous study showed that mucin can increase phage adhesion indirectly by immobilizing host cells [14]. Therefore, we tested the attachment of *B. subtilis* and *V. anguillarum* cells to mucin-coated surfaces. In this experiment, diluted bacterial cultures were transferred to 1.5% agar plates that were left uncoated or coated with PBS or mucin. Bacteria were allowed to attach for 6 hours. Afterwards, a piece of agar with attached cells was cut out, and the cells were detached and analyzed by flow cytometry.

**Fig. 2:**
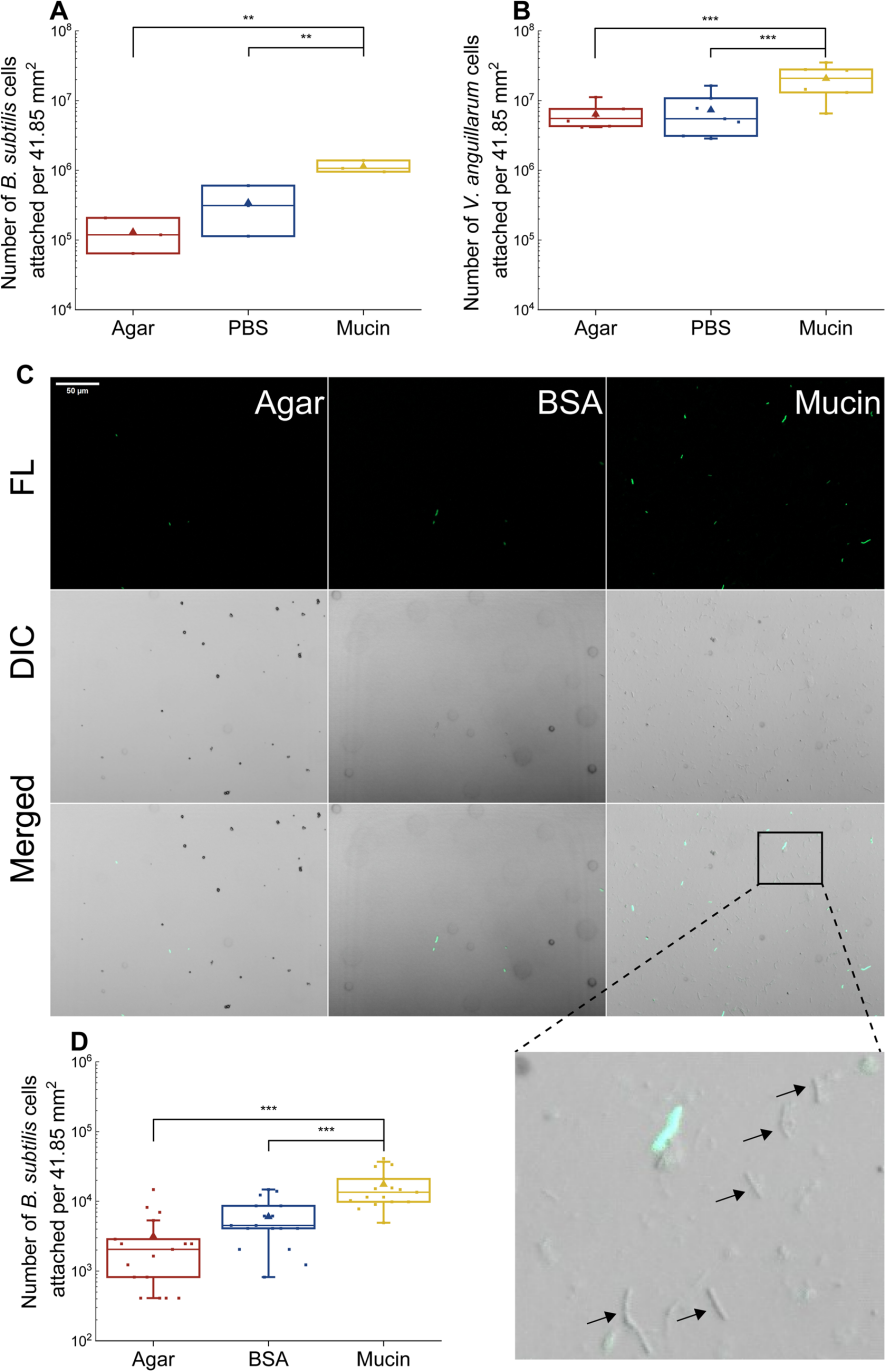
Number of *B. subtilis* and *V. anguillarum* cells attached to different surfaces, including an uncoated agar surface, a PBS-coated surface, a BSA-coated surface, and a mucin-coated surface. Statistical significance was assessed using ANOVA with Tukey’s post hoc test. *,* p* < 0.05; **, *p* < 0.01; ***, *p* < 0.001. (A) Counting of *B. subtilis* cells by flow cytometry after 6 hours of incubation on the plates (n = 3). (B) Counting of *V. anguillarum* cells by flow cytometry after 6 hours of incubation on the plates (n = 6). (C) Fluorescence, DIC, and merged microscopy images of *B. subtilis* cells attached to different surfaces (uncoated 1.5% agar, BSA-coated surface, and mucin-coated surface). Top, fluorescence microscopy images showing GFP-expressing cells (TL); middle, DIC; bottom, merged image. Arrows indicate the adhered cells where the fluorescent signal was obstructed. The scale bar in the upper left corner represents 50 µm. (D) Counting of *B. subtilis* by microscopy after 1 hour of incubation on the plates (n = 15 fields of view).

The data showed that the mean number of *B. subtilis* cells attached to the mucin-coated surface was more than threefold higher than the number attached to the PBS-coated surface (*p* = 0.007), and over eightfold higher than the number attached to the uncoated agar surface (*p* = 0.001) (Fig. 2A). In case of *V. anguillarum,* the number of cells attached to mucin was nearly threefold higher than the number attached to the PBS-coated surface (*p* = 0.002) and more than threefold higher than the number attached to the uncoated agar plates (*p* = 0.001) (Fig. 2B).

In the above experiment, the bacteria on the test surfaces were incubated for 6 hours, potentially allowing them to grow. We therefore used a different attachment test with a shorter exposure time. To do this, we used GFP-tagged *B. subtilis*, which allowed us to count the attached cells using fluorescent microscopy and imaging. Fluorescently labelled *B. subtilis* cells were incubated for 1 hour on the different test surfaces, processed as described above, and examined by confocal microscopy using the differential interference contrast (DIC) and fluorescence modes (Fig. 2C).

The results showed that mucin significantly enhanced the attachment of *B. subtilis* cells to agar (Fig. 2D). The mean number of cells attached to mucin was 2.9 times higher than the number attached to the BSA-coated surface (*p* = 0.000002) and 5.5 times higher than the number attached to the uncoated agar surface (*p* = 0.00008). In addition, it was observed that mucin has an impact on the fluorescence of cells. On surfaces covered with mucin, in contrast to BSA, some cells did not give a fluorescent signal (Fig. 2C), suggesting that mucin either altered the metabolic activity of the cell or physically coated the cell surface, obscuring the fluorescence signal.

### Mucin promotes metabolic activity of surface-attached *Vibrio anguillarum*, but not of *Bacillus subtilis*

**Fig. 3:**
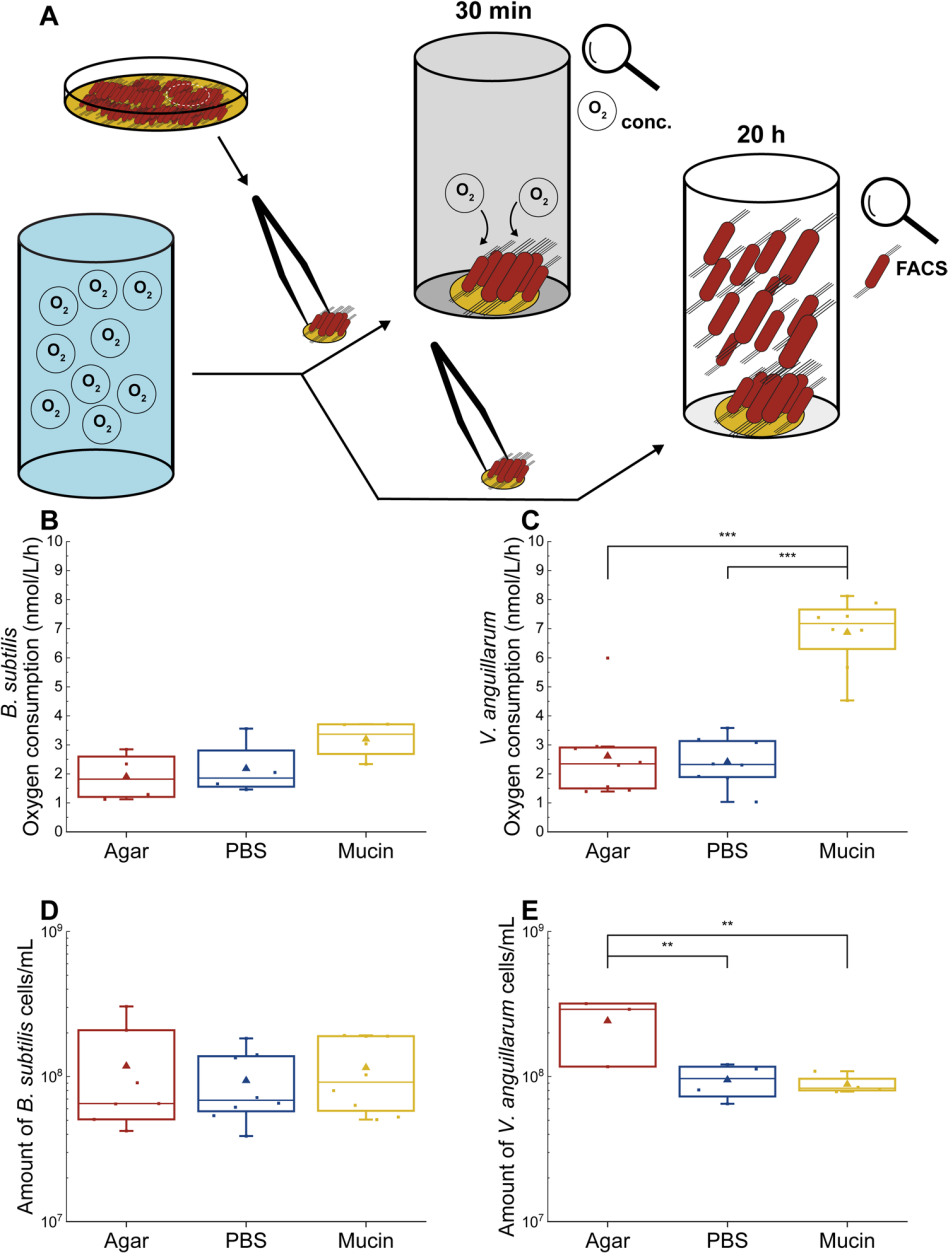
Oxygen consumption rate and regrowth of *B. subtilis* and *V. anguillarum* cells attached to three surfaces: an uncoated agar surface, a PBS-coated surface, and a mucin-coated surface. Statistical significance was assessed using ANOVA with Tukey’s post hoc test. **, *p* < 0.01; ***, *p* < 0.001. (A) Graphical representation of the experiment. (B) Activity of *B. subtilis* cells that were allowed to attach for 6 hours (n = 4). (C) Activity of *V. anguillarum* cells that were allowed to attach for 6 hours (n = 8). (D) Number of regrown *B. subtilis* cells from cells attached to different surfaces (n = 8). (E) Number of regrown *V. anguillarum* cells from cells attached to different surfaces (n = 8).

To evaluate the effect of mucin on the metabolic activity of attached *B. subtilis* and *V. anguillarum* cells, bacterial respiration on different surfaces was quantified. In this experiment, *B. subtilis* cells showed a slight increase in oxygen consumption on mucin-coated surfaces, but this difference was not statistically significant (Fig. 3B). In contrast, *V. anguillarum* cells attached to the mucin-coated surface consumed oxygen at a nearly threefold higher rate compared to cells attached to a PBS-coated surface (*p* = 0.0000001) or an uncoated agar surface (*p* = 0.0000003) (Fig. 3C).

To test whether mucin affected the release of cells from the agar surfaces, we placed the agar slices in liquid medium and quantified the regrowth of *B. subtilis* and *V. anguillarum* cells.

After incubation for 20 h (Fig. 3D), no statistically significant difference was observed with *B. subtilis.* However, surface coating did have a strong impact on the number of *V. anguillarum* cells that were released from the surface into fresh medium. The tubes with uncoated agar pieces showed the highest *V. anguillarum* cell concentration (Fig. 3E), nearly threefold higher than in those with mucin-coated pieces (*p* = 0.003) and in those with PBS-coated pieces (*p* = 0.004).

Overall, the effect of mucin on surface-associated metabolic activity and the retention of cells in the biofilm differed between *B. subtilis* and *V. anguillarum.* While no significant effects were observed for *B. subtilis,* mucin stimulated the metabolic activity of the attached *V. anguillarum* cells and reduced the release of cells from the biofilm to the liquid medium.

### Mucin promotes surface adherence of *Bacillus subtilis*, but not *Vibrio anguillarum*, in the presence of phages

**Fig. 4:**
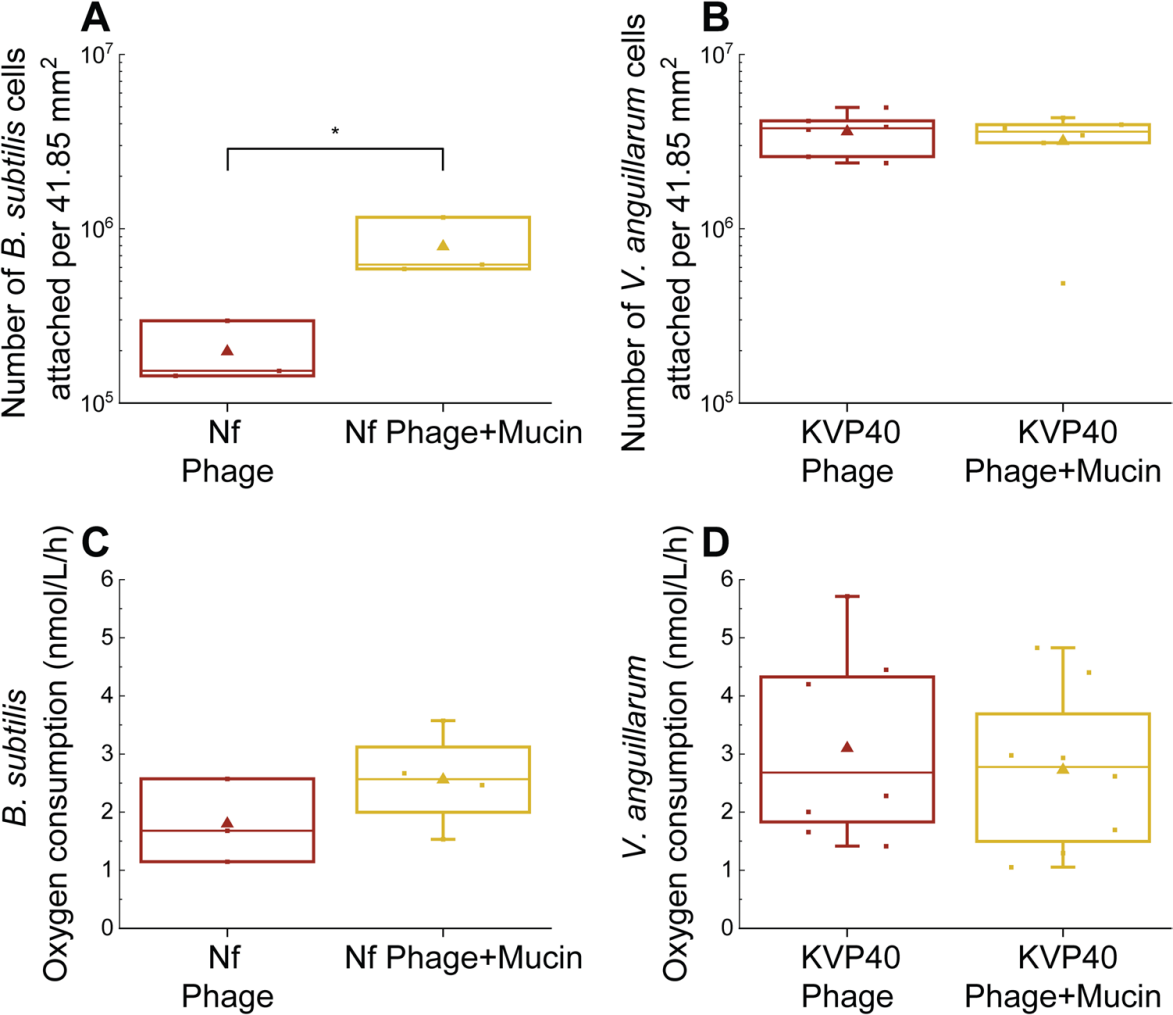
Number of *B. subtilis* and *V. anguillarum* cells attached to surfaces coated with phages only or with mucin + phages, and measurement of their metabolic activity. Statistical significance was assessed using the Tukey test. One star (*) represents statistically significant differences with *p* < 0.05. (A) Counting of *B. subtilis* cells by flow cytometry after 6 hours of incubation on the plates (n = 3). (B) Counting of *V. anguillarum* cells by flow cytometry after 6 hours of incubation on the plates (n = 6). (C) Oxygen consumption rate of *B. subtilis* cells that were attached to phage-coated or mucin+phage-coated surfaces for 6 hours (n = 4). (D) Oxygen consumption rate of *V. anguillarum* cells that were attached to phage-coated or mucin+phage-coated surfaces for 6 hours (n = 8)

To examine whether the adherence of *B. subtilis* and *V. anguillarum* to mucin-coated surfaces is affected by the presence of phages on the surfaces, host cells were exposed to surfaces precoated with phages and mucin, or with phages alone. Interestingly, in the case of *V. anguillarum, the* presence of mucin did not influence the number of attached cells (Fig. 4B), whereas in the presence of phages, mucin still had positive effect on the attachment of *B. subtilis* cells and four times more cells were bound to the mucin+phage-coated surface than to the surface with phages alone (Fig. 4A).

We also measured the metabolic activity of the attached *B. subtilis* and *V. anguillarum* cells through measurement of the oxygen consumption rate. Although we did not detect any statistically significant differences, the metabolic activity of *B. subtilis* appeared to be 1.2-fold higher in the presence of phages and mucin when compared to phages alone (Fig. 4C), suggesting that mucin might nevertheless have a subtle effect on bacterial metabolism under certain conditions, possibly by influencing nutrient availability or stress responses.

These results highlight that mucin’s influence on bacterial attachment does not necessarily result in significant metabolic effects. While *B. subtilis* maintained metabolic activity in the presence of mucin and phages, *V. anguillarum* exhibited stronger metabolic shifts when attached to mucin-coated surfaces (Fig. 3). This underscores the species-specific nature of mucin interactions with bacteria and phages, emphasizing the need to examine these interactions on a case-by-case basis.

## Discussion

Our primary goal in this study was to examine the effect of mucins on adherence and interactions of phages and bacteria beyond the model systems tested in previous studies [12, 14]. To achieve this, we applied a nearly identical experimental setup to compare the influence of mucin on two unrelated bacterial species, with contrasting medical significance, namely *Bacillus subtilis,* a beneficial bacterium, and *Vibrio anguillarum,* an important fish pathogen*.*

Our study revealed that the *Bacillus* and *Vibrio* phages that were examined displayed significantly increased attachment to mucin-coated plates in comparison to uncoated LB agar plates and plates coated with BSA. However, for the *Vibrio* phage KVP40, the amount of attachment was only significantly higher relative to BSA-coated plates (Fig. 1). This observation aligns with the findings of Barr et al. [2013], who found an increased attachment of *E. coli* T4 phage to mucin using a similar assay. More-recent studies from different research groups have also shown that other phages can attach to mucin: for example, *E. coli* phages ES17 and øPNJ-6, *Flavobacterium columnare* phage FCL-2, *Aeromonas* sp. phage V46, *Flavobacterium* sp. phage FL-1, and *Pseudomonas aeruginosa* phages GEC_MRC, GEC_K2, and GEC_PNG3. All of these phages, except ES17 contain Ig-like domains on their capsids [11–13, 37].

The adhesion mechanism by which phages adhere to mucin was first attributed to the specific interaction occurring between the variable immunoglobulin Ig-like domains situated on the capsid of the phages and glycans on the surface of mucin [14]. Phage phi29, a member of the phi29-like phage family related to phage Nf, has a bacterial Ig-like domain 2 (BID_2) in its major head protein [38, 39], and bioinformatic analysis performed using the SMART web tool also showed the presence of a BID_2 domain in the major head protein of phage Nf [36]. If BID_2 engages with glycan molecules to promote adhesion, this might clarify the lack of enhanced attachment to BSA, a protein without attached sugar molecules [40].

On other hand, the fact that our bioinformatic screening did not identify any Ig-like domains in the SP10, SPβ, and KVP40 capsid proteins suggests that the increased attachment of these phages to mucin-coated surface involves other modes of interaction with mucin. It is worth noting that our bioinformatic approach did not account for Ig-like domains encoded in other reading frames, as reported previously [38]. Future analyses incorporating frameshift-aware detection methods may provide further insights into the structural adaptations of these phages.

The ability of phages to adhere to mucins is likely to reflect adaptation to their specific host environments. Different mucins vary in their glycosylation patterns and structure, which may influence phage binding preferences. In this study, we used type III porcine stomach mucin, a well-characterized mucin type that is widely used in bacterial and phage interaction studies, to provide a standardized model for investigating these interactions

In the updated BAM model, it is proposed that the retention of phages in mucosal areas is associated with the mesh-like structure of mucin, which traps phages, rather than specific binding of Ig-like domains to mucin [8]. However, it has been reported that commercial mucins may not fully recapitulate the physical properties of native mucins, including their structural organization, which raises the possibility that additional or alternative mechanisms may contribute to the observed phage attachment [41]. BSA, which was used as a control in our study, is a globular protein that does not contain glycans and does not form a mesh under the conditions that we used, suggesting that it is the specific properties of mucin that enable phage attachment [40, 42].

In line with previous studies [22, 24], our results confirm that mucin enhances surface attachment of *B. subtilis* and *V. anguillarum* (Fig. 3). *B. subtilis* and *V. anguillarum* have been identified within a microenvironment proximal to mucosal surfaces [19, 43]. *Bacillus cereus*, a relative of *B. subtilis*, has been shown to adhere to mucin through a process that is facilitated by flagellin [23]. The identification of a flagellin coding sequence in the genome of *B. subtilis* P9_B1 suggests that its interaction with mucin could also be facilitated by flagellin [27]. It has been shown that *B. subtilis*, in addition to its capacity to bind to mucosal surfaces, shows a capability for competitive exclusion of specific pathogenic bacteria, such as enterotoxic *E. coli* and *Pseudomonas* spp. [22, 44]. The presence of the flagellin A protein in *V. anguillarum* has been shown by Milton et al. to be essential for its virulence [1996]. This protein probably plays a significant role in promoting the attachment of *V. anguillarum* to mucus in the host and its subsequent entry into the host organism. The flagellin proteins are central components of the bacterial flagellum, a whip-like appendage that enables bacterial motility [45]. The flagellum of pathogenic bacteria such as *V. anguillarum*, might serve to anchor the bacterium to mucosal surfaces to initiate an infection. Furthermore, the expression of the *flaA* gene, encoding flagellin A, has been shown to increase in the presence of mucin [46].

Our results also suggest that mucin-coated surfaces have markedly different effects on the metabolic activity of the attached *Bacillus subtilis* and *Vibrio anguillarum* cells. These differences in metabolic activity may provide additional insight into the distinct effects mucins have on these bacterial species in the presence of phages, potentially influencing the outcomes of phage-host interactions.

The enhanced adherence of *V. anguillarum* and *B. subtilis* cells to mucin was also associated with an increased metabolic rate of *V. anguillarum*, whereas *B. subtilis* metabolism was not significantly affected by the presence of mucin. This difference may be related to fact that *V. anguillarum* PF430-3 is a fish pathogen isolated from salmon skin and is likely to be adapted to mucin-rich environments [47], whereas the *B. subtilis* P9_B1 strain was isolated from soil where interaction with mucin is limited [27]. The stimulation of oxygen consumption observed in mucin-associated *V. anguillarum* biofilms could potentially also be ascribed to the differences in biofilm formation by the two bacteria: *V. anguillarum* mainly develop in submerged biofilms, as opposed to the preference of *B. subtilis* for pellicle formation in the water-air interface [46, 48–52], which is not available in the oxygen sensor vials during oxygen measurements. Another possible explanation could be formation of metabolically dormant spores by certain subpopulations of *B. subtilis*. These spores would then require and consume much less oxygen compared to *V. anguillarum* cells, which cannot sporulate [53].

Importantly, the effect of mucin on attachment persisted in the case of *B. subtilis*, even in the presence of phages on the mucin-coated surface. However, this effect was not observed with *V. anguillarum*, where phage lysis of cells on the phage-coated surfaces likely negated the positive effect of mucin on host attachment. Our observation that some bacterial cells exposed to mucin did not exhibit a fluorescent signal (Fig. 2C) suggests that mucin could either alter cell metabolic activity or physically coat the bacterial surface, potentially obscuring the fluorescence signal. This finding aligns with previous reports that mucin can modulate bacterial physiology and virulence and influence phage-host interactions [9, 10, 12]. This was not the case for *V. anguillarum*, where mucin did not affect phage-host interactions. However, *V. anguillarum* PF430-3 has previously been shown to be protected from phage KVP40 infections inside bacterial aggregates/biofilms in the absence of mucin [6].

So far, the recognition receptor for phage Nf has not been identified. A significant proportion of phages that infect Gram-positive bacteria recognize carbohydrates connected through covalent bonds to the peptidoglycan cell wall [54–56]. It is therefore possible that phage Nf recognizes glycan residues present on mucin in a similar manner.

In contrast to *B. subtilis*, mucin did not influence the effect of phage on *V. anguillarum*. This could be attributed to the fact that phage KVP40 uses the outer membrane protein OmpK as a receptor. Unlike mucin, OmpK lacks glycosylated components and differs significantly in structure [57]. Similarly, it has been reported that *E. coli* is also not shielded by mucin in this tripartite interaction [37].

Almeida et al. [11] demonstrated that supplementation of the culture medium used to grow the fish pathogen *F. columnare* with primary mucus from rainbow trout resulted in alterations in bacterial phenotypic traits and increased the susceptibility of the bacterium to phage infection while at the same time increasing its virulence to the fish. This finding, together with our results, underlines the importance of studying each phage-host interaction in mucosal areas separately and emphasizes that different phages and bacteria interact with mucin in different ways.

It will thus be necessary to examine phage-host interactions in multispecies systems in which the effect of mucin on entire microbial communities can be investigated. Such research may have important implications for the development of phage therapy strategies aimed at treating infections in mucosal environments, where probiotic and pathogenic bacteria, together with their respective phages, coexist.

While our study primarily focused on bacterial attachment and phage adherence to mucin-coated surfaces, we acknowledge that other aspects of the effects of mucin, such as phage growth on mucin-treated cells and bacterial utilization of mucin as a carbon source, were not assessed. Previous studies have shown that exposure to mucin can alter bacterial physiology in ways that increase the production of phage progeny [12]. However, our study design emphasized bacterial attachment dynamics and metabolic activity on surfaces, rather than planktonic growth and phage production. Future studies could complement the current one by evaluating whether mucin-induced physiological changes influence phage replication dynamics.

It remains to be determined whether *B. subtilis* or *V. anguillarum* actively utilizes mucin components for growth. Although mucin was primarily used as a structural matrix in this study, studies on its role as a nutrient source could provide a more comprehensive view of mucin’s multifaceted impact on bacterial physiology and phage-host interactions.

## Conclusion

Building upon the groundwork laid by Barr et al. in 2013 with the BAM model, our study has expanded the scope of that investigation to previously untested lytic and temperate phages. We observed that all of the *Bacillus* phages tested were more likely to attach to surfaces if they were coated with mucin. Furthermore, the bacterial species *B. subtilis* and *V. anguillarum* also exhibited a significant increase in attachment to mucin-coated surfaces, in agreement with previous observations. Surprisingly, however, mucin seemed to promote attachment of *B. subtilis*, but not of *V. anguillarum*, when the surface was pre-coated with phages. This could indicate that mucin shields certain bacteria, preventing successful phage infection. This suggests potential applications for influencing probiotic interactions and competitive exclusion of pathogens. While both bacterial species exhibited increased attachment to mucin-coated surfaces, *V. anguillarum* showed decreased attachment when phages were present. This novel finding suggests that phages could play a role in modulating bacterial attachment to mucins, with potential implications for the phage-bacteria dynamics of mucosal communities. Expanding this research to encompass a broader range of bacterial species and phage families would enhance our understanding of the universality of these interactions and could potentially lead to the development of strategies to manipulate microbial communities in various settings, including medical and industrial applications. In conclusion, this study has revealed new information about interactions between mucins, bacteria, and phages. These findings provide a foundation for future research aimed at understanding the dynamics of microbial communities within mucosal environments.

## Supplementary Information

Below is the link to the electronic supplementary material.

Supplementary file1 (DOCX 273 KB)

## Data Availability

All raw data are available from the corresponding author upon request.
