# Supplementary material for: Distinct effects of mucin on phage-host interactions in model systems of beneficial and pathogenic bacteria
Source: Arch Virol. 2025 May 20;170(6):133. doi: 10.1007/s00705-025-06322-5 (PMC12092537; doi:10.1007/s00705-025-06322-5)
Supplement: Supplementary file 1 — Supplementary file1 (DOCX 273 KB) [file 705_2025_6322_MOESM1_ESM.docx]

Supplemental data 1

Macro used for counting the cells in the images from the microscope in Fiji (ImageJ) software.

// Set the input and output directories

inputDir = "inputDir";

outputDir = "outputDir";

// Set the file extension of the images (e.g., "tif", "jpg", "png", etc.)

fileExtension = "czi";

// Get the list of image files in the input folder

list = getFileList(inputDir);

// Load necessary plugins (only required if not already installed)

run("Blobs (25K)");

run("Bio-Formats Macro Extensions");

// Loop through the list of files

for (i = 0; i < list.length; i++) {

if (endsWith(list[i], fileExtension)) {

// Open the image

open(inputDir + list[i]);

// Process the image

run("Convert to Mask");

// Adjust the threshold if necessary

setAutoThreshold("Default");

// Run the "Analyze Particles" function

run("Analyze Particles...", "size=0-Infinity show=Overlay summarize add");

// Save the results to a CSV file

saveAs("Results", outputDir + File.nameWithoutExtension + "_results.csv");

// Close the image

close();

}

}

// Notify when the processing is complete

print("Cell counting for all images in the folder is complete.");


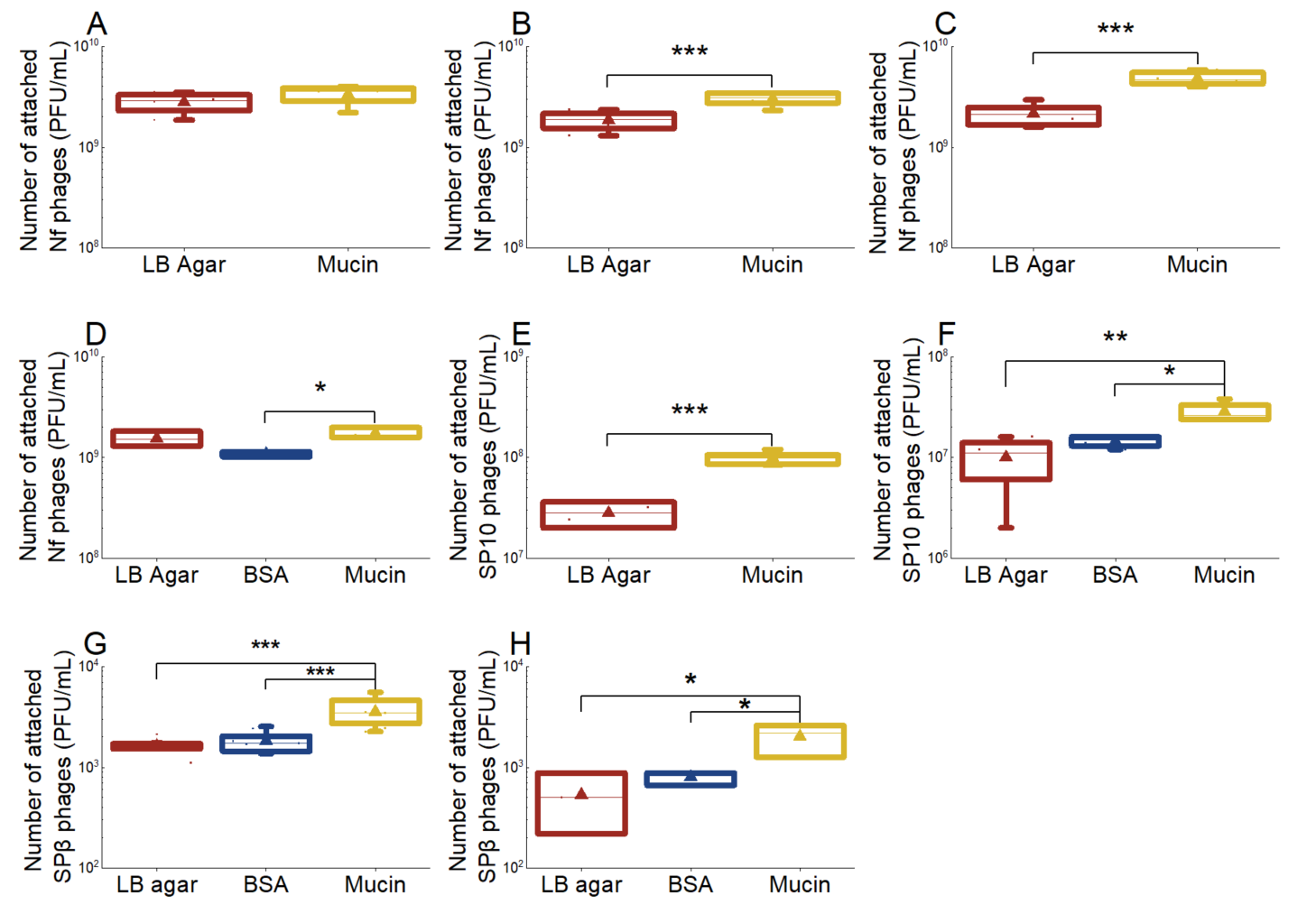


**Supplemental figure 1**: Data from all individual experiments of *Bacillus* phages attached to mucin prior to normalization. A, B, C and D represent PFU / mL of Nf phage attached, E and F represent PFU / mL of SP10 phage attached and G and H represent PFU / mL of SPβ phage attached. Each letter represents an independent run of the same type of experiment.
